# Pancreatic **β** cell–selective zinc transporter 8 insufficiency accelerates diabetes associated with islet amyloidosis

**DOI:** 10.1172/jci.insight.143037

**Published:** 2021-05-24

**Authors:** Jie Xu, Nadeeja Wijesekara, Romario Regeenes, Dana Al Rijjal, Anthony L. Piro, Youchen Song, Anne Wu, Alpana Bhattacharjee, Ying Liu, Lucy Marzban, Jonathan V. Rocheleau, Paul E. Fraser, Feihan F. Dai, Cheng Hu, Michael B. Wheeler

**Affiliations:** 1 Shanghai Diabetes Institute, Shanghai Key Laboratory of Diabetes Mellitus, Shanghai Key Clinical Center for Metabolic Diseases, Shanghai Jiao Tong University Affiliated Sixth People’s Hospital, Shanghai, China.; 2 Department of Physiology, University of Toronto, Toronto, Ontario, Canada.; 3 Tanz Centre for Research in Neurodegenerative Diseases, University of Toronto, Toronto Western Hospital, Toronto, Ontario Canada.; 4 Institute of Biomaterials and Biomedical Engineering, University of Toronto, Toronto, Ontario, Canada.; 5 Division of Advanced Diagnostics, Toronto General Hospital Research Institute, Toronto General Hospital, Toronto, Ontario, Canada.; 6 College of Pharmacy, Rady Faculty of Health Sciences, University of Manitoba, Winnipeg, Manitoba, Canada.; 7 Toronto General Research Institute, University Health Network, Toronto, Ontario, Canada.; 8 Department of Medical Biophysics, University of Toronto, Toronto, Ontario, Canada.; 9 Institute for Metabolic Disease, Fengxian Central Hospital Affiliated to Southern Medical University, Shanghai, China.

**Keywords:** Endocrinology, Metabolism, Beta cells, Diabetes, Insulin

## Abstract

GWAS have shown that the common R325W variant of *SLC30A8* (ZnT8) increases the risk of type 2 diabetes (T2D). However, ZnT8 haploinsufficiency is protective against T2D in humans, counterintuitive to earlier work in humans and mouse models. Therefore, whether decreasing ZnT8 activity is beneficial or detrimental to β cell function, especially under conditions of metabolic stress, remains unknown. In order to examine whether the existence of human islet amyloid polypeptide (hIAPP), a coresident of the insulin granule, affects the role of ZnT8 in regulating β cell function, hIAPP-expressing transgenics were generated with reduced ZnT8 (ZnT8B^+/–^ hIAPP) or null ZnT8 (ZnT8B^–/–^ hIAPP) expression specifically in β cells. We showed that ZnT8B^–/–^ hIAPP mice on a high-fat diet had intensified amyloid deposition and further impaired glucose tolerance and insulin secretion compared with control, ZnT8B^–/–^, and hIAPP mice. This can in part be attributed to impaired glucose sensing and islet cell synchronicity. Importantly, ZnT8B^+/–^ hIAPP mice were also glucose intolerant and had reduced insulin secretion and increased amyloid aggregation compared with controls. These data suggest that loss of or reduced ZnT8 activity in β cells heightened the toxicity induced by hIAPP, leading to impaired β cell function and glucose homeostasis associated with metabolic stress.

## Introduction

Zinc ions are highly concentrated in insulin secretory granules where they complex with insulin to form insulin crystals (1, 2). ZnT8 is the primary zinc transporter responsible for the uptake of zinc into these granules. A number of studies of patients with type 2 diabetes (T2D) from different populations have confirmed an association between a nonsynonymous polymorphism in *SLC30A8*, which encodes ZnT8, and elevated disease risk (3–11). The major allele Arg325Trp variant, which is also a risk allele, was associated with increased fasting plasma glucose, reduced insulin secretion, and impaired conversion of proinsulin to insulin (12–14). Global ZnT8-KO mice show reduced islet zinc content, abnormal insulin secretory granule morphology with less insulin crystallization, and impaired glucose tolerance (15–17). Similarly, β cell–specific ZnT8 knock out (ZnT8B-KO) mice also show reduced insulin crystallization and impaired glucose tolerance (18, 19). Additionally, Tamaki et al. reported decreased plasma insulin in ZnT8B-KO mice resulting from enhanced hepatic clearance of insulin (20). We also showed that during metabolic stress induced by a high-fat diet (HFD), global ZnT8-KO mice developed severe insulin resistance and obesity, whereas mice with specific deletion of ZnT8 in β cells did not (19).

Counterintuitive to these studies on reduced ZnT8 activity, Flannick et al. reported 12 rare *SLC30A8*/ZnT8 loss-of-function variants present in approximately 350 of 150,000 genotyped individuals where haploinsufficiency was observed to statistically reduce T2D risk (21). One of these mutations, *SLC30A8* R138*, was associated with lower rates of T2D and increased insulin secretion in humans (21). Mice carrying *SLC30A8* R138* showed a significant increase in insulin secretion with no changes to glucose and insulin tolerance (22, 23), implying that decreased β cell ZnT8 activity reduces T2D risk. This contrasts with several reports that previously showed a relationship between ZnT8 activity and T2D risk centered on p.Arg325Trp, where reduced zinc transport was associated with impaired β cell function and increased T2D risk (15, 24). However, Merriman et al. reported that the Arg325 risk variant has higher zinc transport activity than the Trp325 variant (25), whereas Carvalho et al. showed there is no difference of zinc transport activity between Trp325 and Arg325 variants (26). Thus, whether decreasing ZnT8 activity is beneficial or detrimental to β cell function indeed remains an open question. If we are to completely understand the functional role of ZnT8, it is imperative to look at its impact on another important resident of the insulin granule, islet amyloid polypeptide (IAPP), an important contributor to T2D. IAPP, or amylin, is an amyloidogenic protein that is cosecreted with insulin. IAPP aggregates in human islets and is a common finding in autopsies of patients with T2D. Since IAPP does not aggregate in rodents, transgenic rodent models expressing human IAPP (hIAPP) have been developed (27). Mice homogenous for hIAPP spontaneously develop diabetes due to β cell death, impaired insulin secretion, and hyperglycemia (28). Hemizygotes can also develop diabetes when on a HFD (29).

In the present study, we sought to determine whether reducing or eliminating ZnT8 activity specifically in β cells improves or impairs cell function in the presence of hIAPP. To test this, mice expressing hIAPP were crossed with mice with reduced or null ZnT8 expression specifically in β cells. We demonstrated that under metabolic stress, these animals had heightened glucose intolerance, islet amyloid deposition, and impaired insulin secretion.

## Results

### Absence of ZnT8 exacerbates glucose intolerance in hIAPP-transgenic mice.

**Figure 1:**
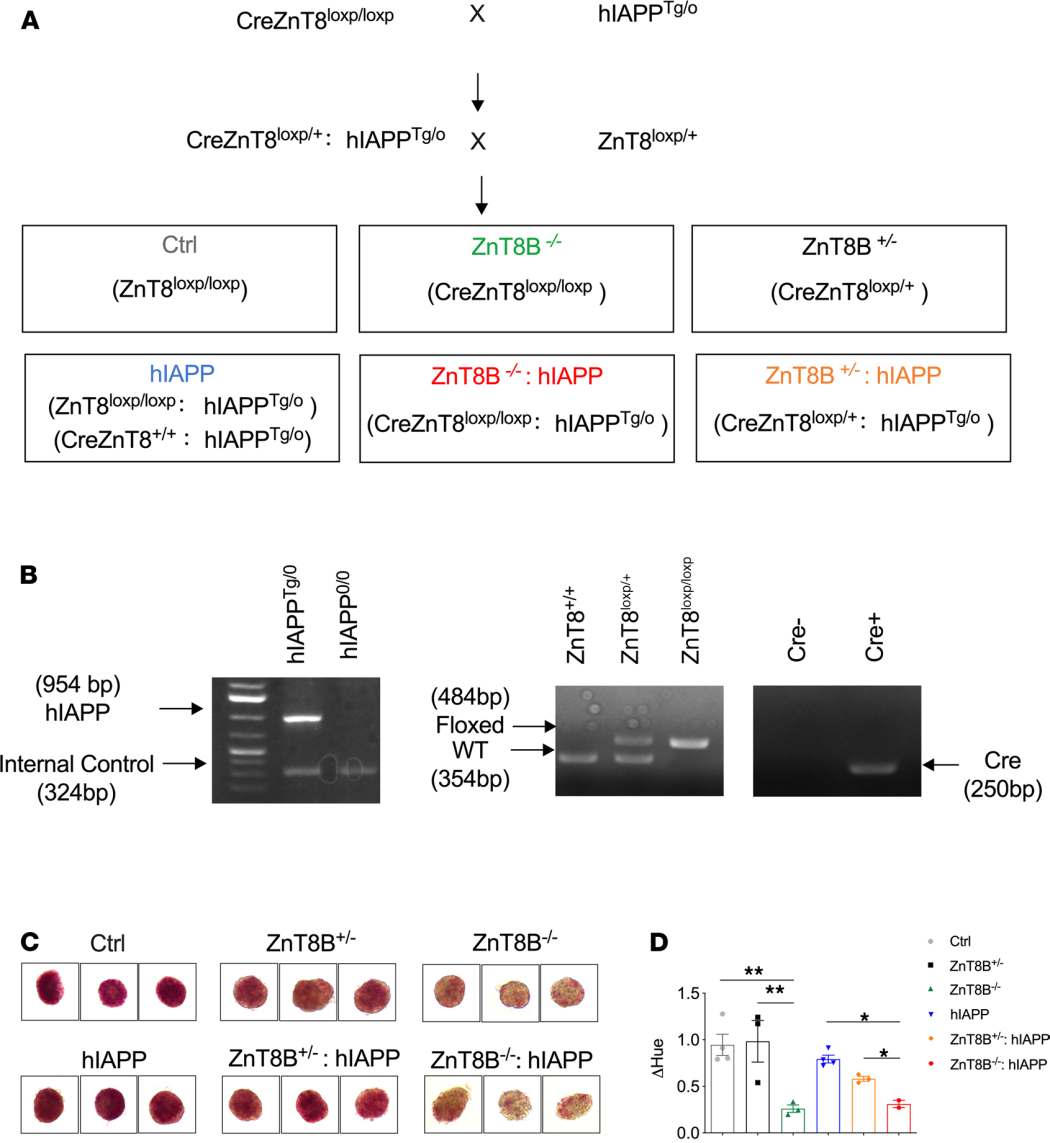
Generation of ZnT8B^–/–^ hIAPP and ZnT8B^+/–^ hIAPP mice. (**A**) Breeding and (**B**) genotyping strategy used to identify ZnT8B^–/–^ hIAPP and ZnT8B^+/–^ hIAPP mice. (**C**) Dithizone staining and (**D**) its quantification on isolated pancreatic islets from control (*n* = 4), ZnT8B^+/–^ (*n* = 3), ZnT8B^–/–^ (*n* = 3), hIAPP (*n* = 4), ZnT8B^–/–^ hIAPP (*n* = 2), and ZnT8B^+/–^ hIAPP mice (*n* = 3). *P < 0.05, ZnT8B^+/–^ hIAPP or hIAPP groups compared with ZnT8B^–/–^ hIAPP; **P < 0.01, control or ZnT8B^+/–^ groups compared with ZnT8B^–/–^. One-way ANOVA with Tukey’s adjustment was used in **D**.

Mice were generated and 6 groups of mice were characterized based on their genotype. Breeding schemes to generate the desired mouse lineages and genotyping strategies are shown (Figure 1, A and B). ZnT8^loxp/loxp^ mice were used as controls because we observed no differences in glucose tolerance between Cre-positive control mice and ZnT8^loxp/loxp^ controls at 6 weeks of age (Supplemental Figure 1A; supplemental material available online with this article; https://doi.org/10.1172/jci.insight.143037DS1). This is supported by a number of studies showing that Cre expression has no impact on glucose homeostasis in mice (30, 31). We also measured human growth hormone (hGH) expression in islets since rat insulin 2 promoter–Cre (RIP-Cre) mice have been reported to show various β cell abnormalities owing to the presence of this mini gene. We showed confidently with *n* = 4 that hGH levels in all 4 groups of animals were extremely low, well below the sensitivity of the assay (dotted line, Y = 5 pg/mL) of the hGH ELISA kit (Supplemental Figure 1, F and G). This strongly suggests that there was no hGH protein in islets in all 4 genotypes tested. Furthermore, measurement of total insulin content showed similar levels, all within the normal range, in all 4 genotypes (Supplemental Figure 1H), further reiterating the absence of β cell abnormalities owing to the presence of the hGH mini gene. Most importantly, in the present study, we characterized hIAPP mice in the presence or absence of Cre on a HFD for 8–10 weeks (Supplemental Figure 1, B–E). Body weight, 5-hour fasted blood glucose, oral glucose tolerance, and insulin secretion were assessed, and we did not observe any differences between these 2 groups.

Pancreatic β cell–specific ZnT8 deletion resulted in reductions in intracellular zinc levels in islets as determined by dithizone staining (Figure 1, C and D). Quantification of dithizone staining was performed by converting the RGB value to Hue, which showed that there were significantly reduced zinc levels associated with homozygous KO of ZnT8 compared with control and hIAPP mice.

**Figure 2:**
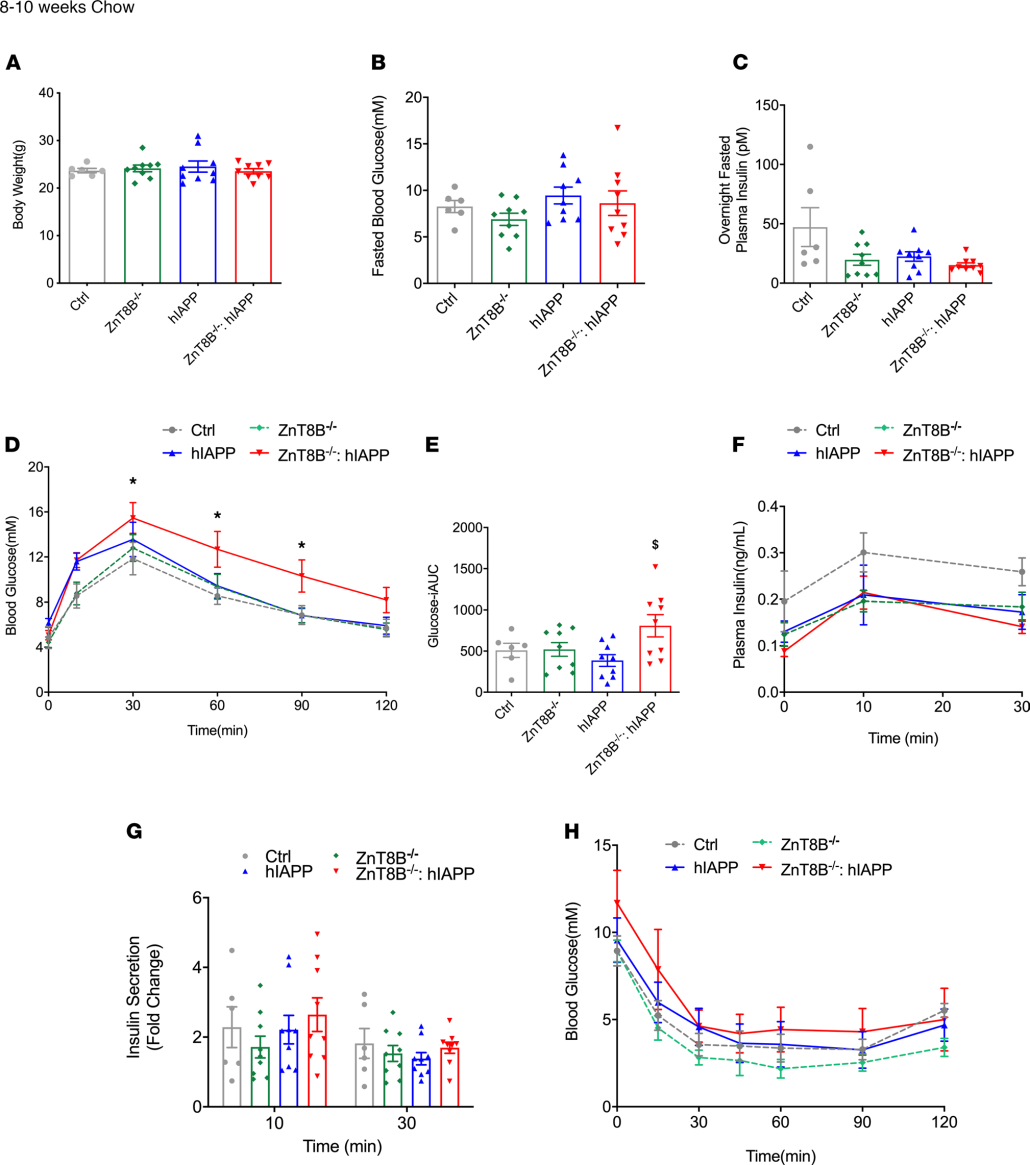
Chow-fed ZnT8B^–/–^ hIAPP mice show significantly impaired glucose tolerance. (**A**) Body weight, (**B**) 5-hour fasted blood glucose, (**C**) overnight fasted plasma insulin, (**D**) oral glucose tolerance test (OGTT), and (**E**) incremental area under the glucose curve (iAUC). (**F**) Plasma insulin during OGTT with (**G**) in vivo glucose-stimulated insulin secretion expressed as fold change over basal. (**H**) Insulin tolerance test (ITT). *P < 0.05, all groups compared with control. ^$^*P* < 0.05, all groups compared with hIAPPs. Data are represented as mean ± SEM. (**A**–**H**) Control (*n* = 6), ZnT8B^–/–^ (*n* = 9), hIAPP (*n* = 9), and ZnT8B^–/–^ hIAPP (*n* = 9). One-way ANOVA with Tukey’s adjustment was used in **A**–**C** and **E**. Two-way ANOVA with Dunnett’s correction was used in **D**, **F**, **G**, and **H**.

Male mice of all 4 genotypes after 8–10 weeks of chow diet feeding exhibited similar body weight, 5-hour fasted blood glucose, and overnight fasted plasma insulin (Figure 2, A–C). ZnT8B^–/–^ hIAPP mice were glucose intolerant compared with controls and hIAPPs (Figure 2, D and E). However, there was no significant impact on insulin secretion or sensitivity (Figure 2, F–H). By 20–22 weeks, hIAPP mice without ZnT8 remained glucose intolerant compared with controls, although hIAPP mice with or without ZnT8 in β cells showed similar impairments in insulin secretion (Supplemental Figure 2, D and E). Body weight, fasting blood glucose, and fasting plasma insulin showed no difference between groups at this age (Supplemental Figure 2, A–C). These data suggest that absence of ZnT8 in β cells had a negative metabolic impact on hIAPP-expressing transgenic mice.

**Figure 3:**
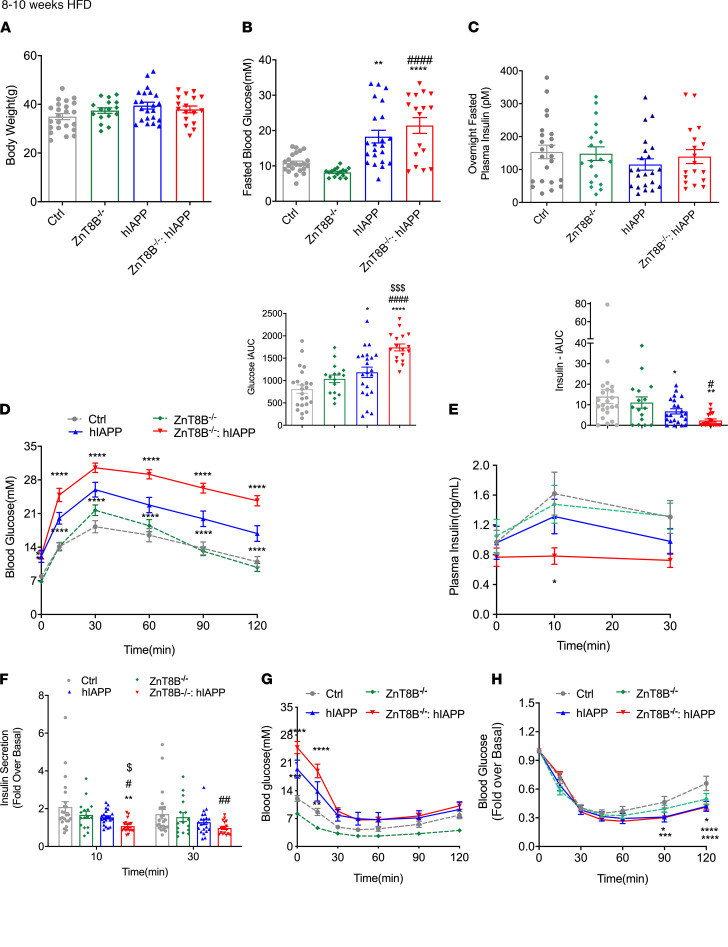
ZnT8B^–/–^ hIAPP mice fed a high-fat diet show impaired glucose tolerance and insulin secretion in vivo. (**A**) Body weight, (**B**) 5-hour fasted blood glucose, (**C**) overnight fasted plasma insulin, (**D**) OGTT and incremental area under the glucose curve, (**E**) plasma insulin during OGTT and incremental area under the insulin curve, with (**F**) glucose-stimulated insulin secretion expressed as fold change over basal. (**G**) Insulin tolerance test (ITT) with (**H**) expression as fold change over basal. Data are represented as mean ± SEM. (**A**–**H**) Control (*n* = 23), ZnT8B^–/–^ (*n* = 16), hIAPP (*n* = 22), and ZnT8B^–/–^ hIAPP (*n* = 17). One-way ANOVA with Tukey’s adjustment was used in **A**–**E**. Two-way ANOVA with Dunnett’s correction was used in **D**–**H**. *P < 0.05, **P < 0.01, ***P < 0.001, ****P < 0.0001, all groups compared with control. **^#^***P* < 0.05, **^##^***P* < 0.01, **^####^***P* < 0.0001, all groups compared with ZnT8B^–/–^. ^$^*P* < 0.05, ^$$$^*P* < 0.001, ^$$$$^*P* < 0.001, all groups compared with hIAPP.

In order to understand the response to metabolic stress, mice were fed a 60% HFD for 8–10 weeks. All male mice showed similar body weight at 8–10 weeks (Figure 3A). ZnT8B^–/–^ hIAPP and hIAPP mice showed fasting hyperglycemia (Figure 3B) and were more glucose intolerant compared with control mice, as seen during an OGTT (Figure 3D). Further, ZnT8B^–/–^ hIAPP mice were significantly more glucose intolerant compared with hIAPP and ZnT8B^–/–^ mice as indicated by the AUC (Figure 3D). They also showed a significant reduction in insulin secretion during the OGTT compared with all 3 controls (Figure 3, E and F). Plasma insulin measurements after an overnight fast showed no significant differences between groups (Figure 3C). Response to insulin injection was also similar in all groups (Figure 3, G and H). By 20–22 weeks of HFD feeding, hIAPP and ZnT8B^–/–^ hIAPP mice were similarly hyperglycemic and glucose intolerant and showed impaired insulin secretion during an OGTT (Supplemental Figure 2, G, I, and J). Body weight and fasting plasma insulin remained similar between all groups (Supplemental Figure 2, F and H). These data further suggest that absence of ZnT8 exacerbated glucose dysregulation induced by metabolic stress in hIAPP mice; however, the differences between hIAPP mice with or without ZnT8 in β cells became negligible as they aged.

### Absence of ZnT8 leads to increased amyloid accumulation, reduced β cell area, and altered β cell morphology.

**Figure 4:**
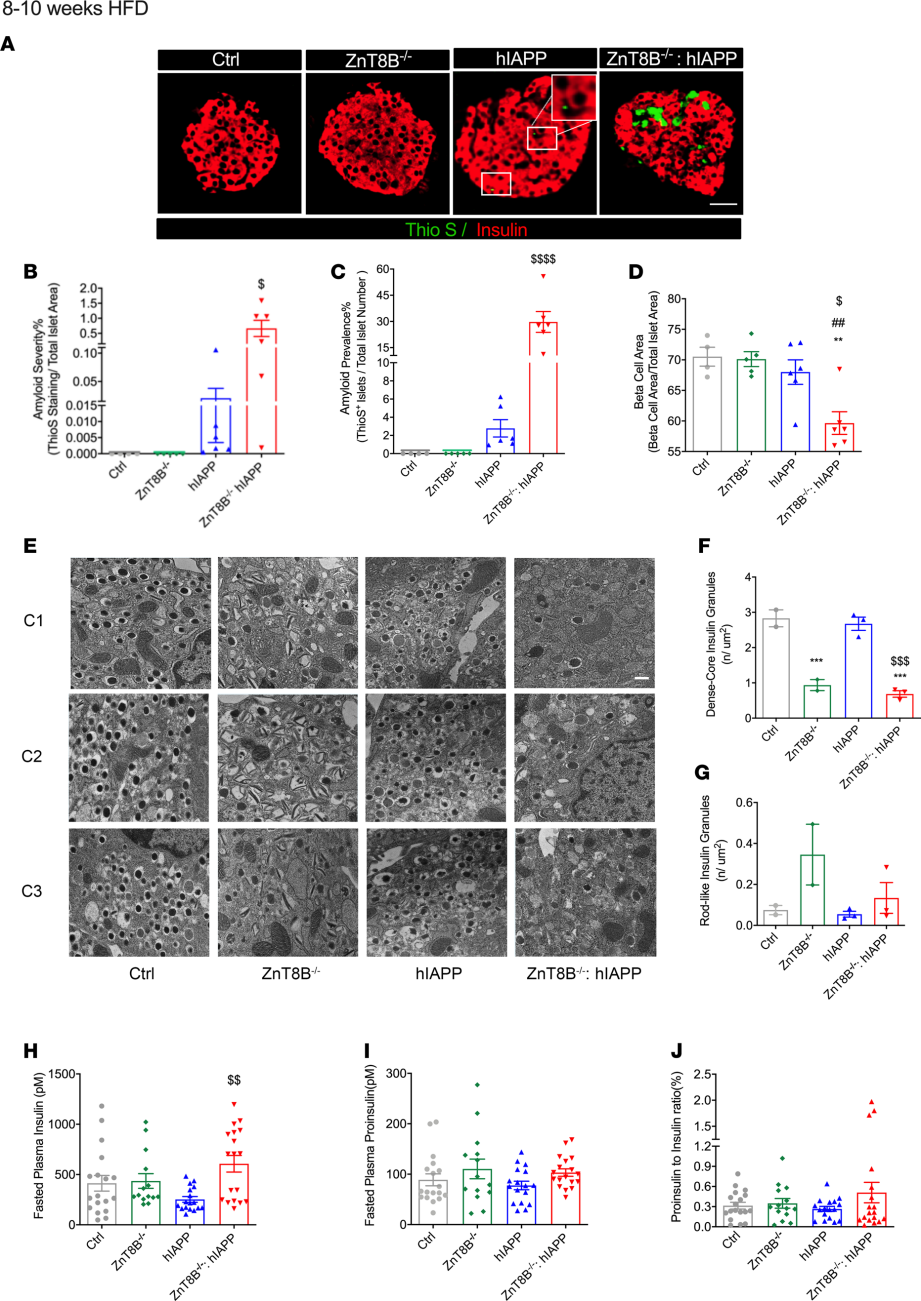
ZnT8B^–/–^ hIAPP mice have increased islet amyloidogenesis, reduced β cell area, and abnormal β cell morphology. (**A**) Thioflavin S staining (scale bar: 50 μm) with quantification of (**B**) amyloid severity, (**C**) amyloid prevalence, and (**D**) β cell area. (**E**) Transmission electron microscopy on isolated islets (animal number, *n* = 2–3/genotype, scale bar: 500 nm), representative cell 1 (C1), cell 2 (C2), and cell 3 (C3). (**F**) Quantification of the number of dense-core granules and (**G**) rod-like insulin granules. (**H**) Five-hour fasted plasma insulin, (**I**) 5-hour fasted plasma proinsulin, and (**J**) proinsulin to insulin ratio. Data are represented as mean ± SEM. **P < 0.01, ***P < 0.001, all groups compared with control, **^##^***P* < 0.01, all compared with ZnT8B^–/–^, ^$^*P* < 0.05, ^$$^*P* < 0.01, ^$$$^*P* < 0.001, ^$$$$^*P* < 0.0001, all groups compared with hIAPP. (**A**–**D**) Control (*n* = 4), ZnT8B^–/–^ (*n* = 5), hIAPP (*n* = 6), and ZnT8B^–/–^ hIAPP (*n* = 6). (**H**–**J**) Control (*n* = 18), ZnT8B^–/–^ (*n* = 14), hIAPP (*n* = 17), and ZnT8B^–/–^ hIAPP (*n* = 18). One-way ANOVA with Tukey’s adjustment was used in **B**–**D** and **F**–**J**.

To determine the effects of lack of ZnT8 in β cells on islet amyloid deposition, we stained pancreatic sections with thioflavin S. After 8–10 weeks of HFD, ZnT8B^–/–^ hIAPP mice compared with hIAPP controls showed significantly elevated amyloid severity measured as the total amyloid per islet area and amyloid prevalence measured as the number of islets positive for amyloid staining (Figure 4, A–C). β Cell area analyzed by insulin per total islet area was reduced by 10% in ZnT8B^–/–^ hIAPP islets compared with all 3 control groups (Figure 4D). Islet morphology analysis by insulin staining (Supplemental Figure 3A) and glucagon staining (Supplemental Figure 3, F and G) revealed no significant differences in islet number (Supplemental Figure 3B), islet size (Supplemental Figure 3C), islet density (Supplemental Figure 3D), or insulin-positive cell area (Supplemental Figure 3E).

After 8–10 weeks of HFD feeding, β cell morphology was significantly altered in ZnT8B^–/–^ hIAPP mice compared with controls (Figure 4E). The number of dense-core insulin granules per area was similarly reduced in ZnT8B^–/–^ and ZnT8B^–/–^ hIAPPs (Figure 4F). Although a significant increase in rod-like granules was observed in ZnT8B^–/–^ mice compared with control mice (Figure 4G), ZnT8B^–/–^ hIAPP mice showed no change in rod-like granules, suggesting that overall, there was a reduction in the total number of granules in these islets compared with ZnT8B^–/–^. Five-hour fasted plasma insulin levels were significantly elevated in ZnT8B^–/–^ hIAPP mice compared with hIAPP mice; there was a trend toward an increase in proinsulin levels and proinsulin/insulin ratio in plasma at 8–10 weeks (Figure 4, H–J). Cumulatively, these data showed an exacerbation of amyloid aggregation in the absence of ZnT8 leading to β cell loss.

### Absence of ZnT8 worsens pancreatic β cell function in hIAPP mice, associated with impaired glucose sensing and cell synchronicity.

**Figure 5:**
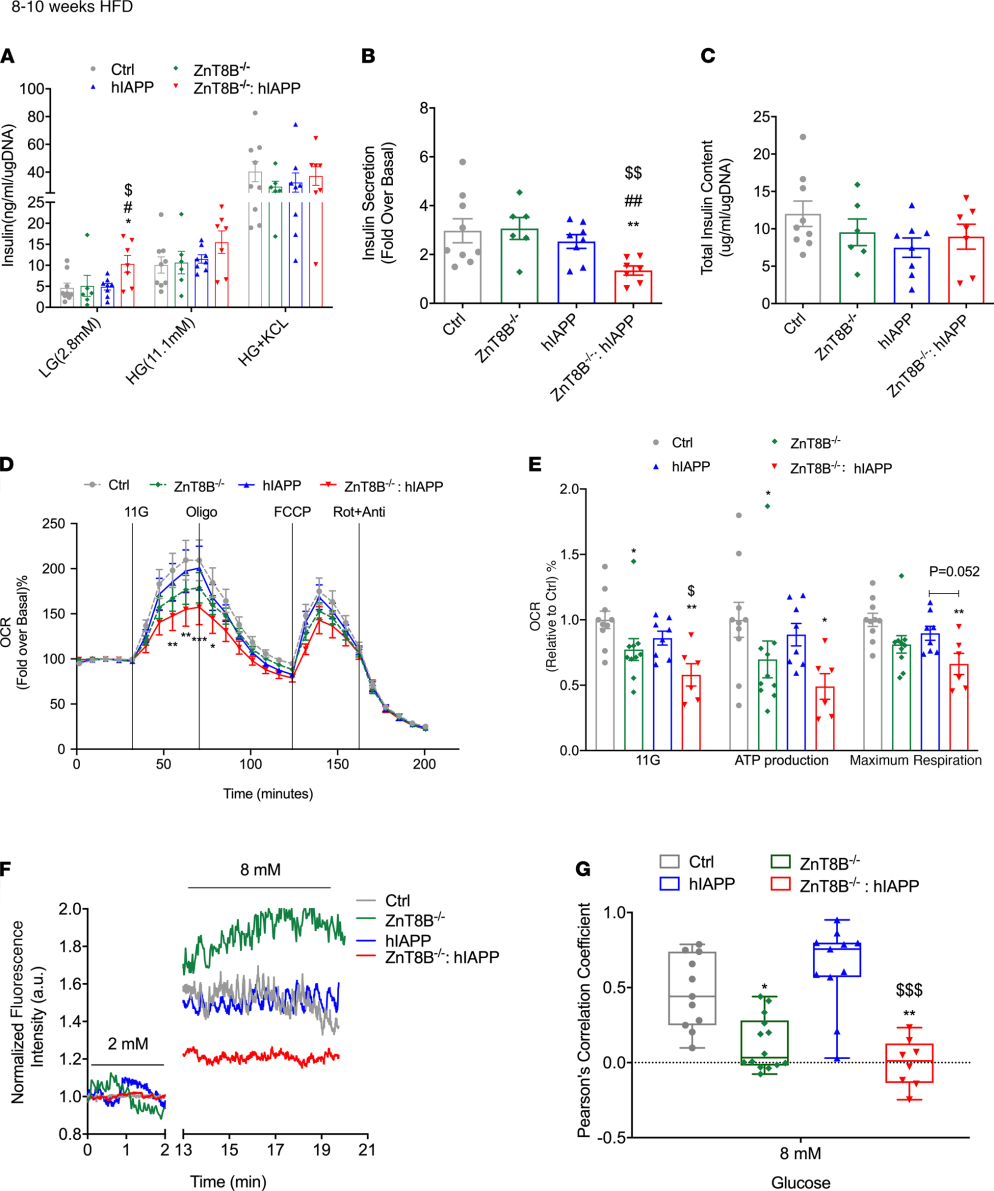
ZnT8B^–/–^ hIAPP mice show impaired insulin secretion, glucose sensing, and cell synchronicity ex vivo. (**A**) Glucose-stimulated insulin secretion from isolated islets with (**B**) glucose-stimulated insulin secretion expressed as fold change over basal, (**C**) total islet insulin content. (**D**) Oxygen consumption rate (OCR) measurement was acquired by Seahorse metabolic flux analyzer and (**E**) glucose-stimulated respiration and mitochondrial functional parameters were quantified as a fold change relative to controls. (**F**) Representative Ca^2+^ trace of control, ZnT8B^–/–^, hIAPP, and ZnT8B^–/–^ hIAPP islet cells. Normalized regions of interest of the chosen cell’s cytoplasm to the mean of the time series, and calculated Pearson’s correlation coefficient of islet β cells synchronicity under 8 mM glucose-stimulated condition (**G**). Data are represented as mean ± SEM. (**A**–**C**) Control (*n* = 9), ZnT8B^–/–^ (*n* = 6), hIAPP (*n* = 8), and ZnT8B^–/–^ hIAPP (*n* = 7). (**D** and **E**) Control (*n* = 10), ZnT8B^–/–^ (*n* = 10), hIAPP (*n* = 8), and ZnT8B^–/–^ hIAPP (*n* = 6). (**F** and **G**) Two or 3 islets per mouse, and control (*n* = 6), ZnT8B^–/–^ (*n* = 5), hIAPP (*n* = 6), and ZnT8B^–/–^ hIAPP (*n* = 4). *P < 0.05, **P < 0.01, ***P < 0.001, all groups compared with control. **^#^***P* < 0.05, **^##^***P* < 0.01, all compared with ZnT8B^–/–^. ^$^*P* < 0.05, ^$$^*P* < 0.01, ^$$$^*P* < 0.001, all compared with hIAPP. One-way ANOVA with Tukey’s adjustment was used in **A**–**C**, **E**, and **G**. Two-way ANOVA with Dunnett’s correction was used in **D**.

After 8–10 weeks of HFD feeding, ZnT8B^–/–^ hIAPP isolated pancreatic islets showed exaggerated insulin secretion when stimulated with low glucose (Figure 5A) and a significant attenuation of the fold change of insulin secretion in response to high glucose stimulation (Figure 5B) compared with the 3 controls. There were no differences in KCl-induced insulin secretion (Figure 5A) or in total islet insulin content (Figure 5C). The oxygen consumption rate (OCR) was measured from islets of mice fed a HFD for 8–10 weeks. We observed a significant reduction of the OCR in ZnT8B^–/–^ hIAPP islets compared with control and hIAPP islets (Figure 5D). ZnT8B^–/–^ hIAPP islets had curbed glucose-induced oxygen consumption and reduced ATP production in line with reduced glucose metabolism compared with control and hIAPP islets (Figure 5E). Interestingly, ZnT8B^–/–^ islets also showed reduced glucose-stimulated respiration and maximum respiration, although not to the same extent as ZnT8B^–/–^ hIAPP islets.

Next, we performed Ca^2+^ imaging of intact islets isolated from mice fed a HFD for 8–10 weeks. Electrical oscillations in islet β cells from HFD-fed mice were supported by the demonstration of mouse islet calcium oscillations in response to 8 mM glucose (Figure 5F). Interestingly, ZnT8B^–/–^ hIAPP islets had significantly dampened glucose-dependent Ca^2+^ influx and cell-to-cell synchrony in response to Ca^2+^ influx compared with hIAPP and control islets (Figure 5, F and G), suggesting a loss of coupling between islet cells and/or defective glucose metabolism. Similarly, ZnT8B^–/–^ islet cells also showed impaired cell-to-cell synchrony in Ca^2+^ influx compared with control cells (Figure 5G). Cumulatively, this suggests that absence of ZnT8 caused defects in glucose sensing and cell synchronicity.

### Heterozygous β cell–specific ZnT8-KO mice show significantly impaired insulin secretion.

**Figure 6:**
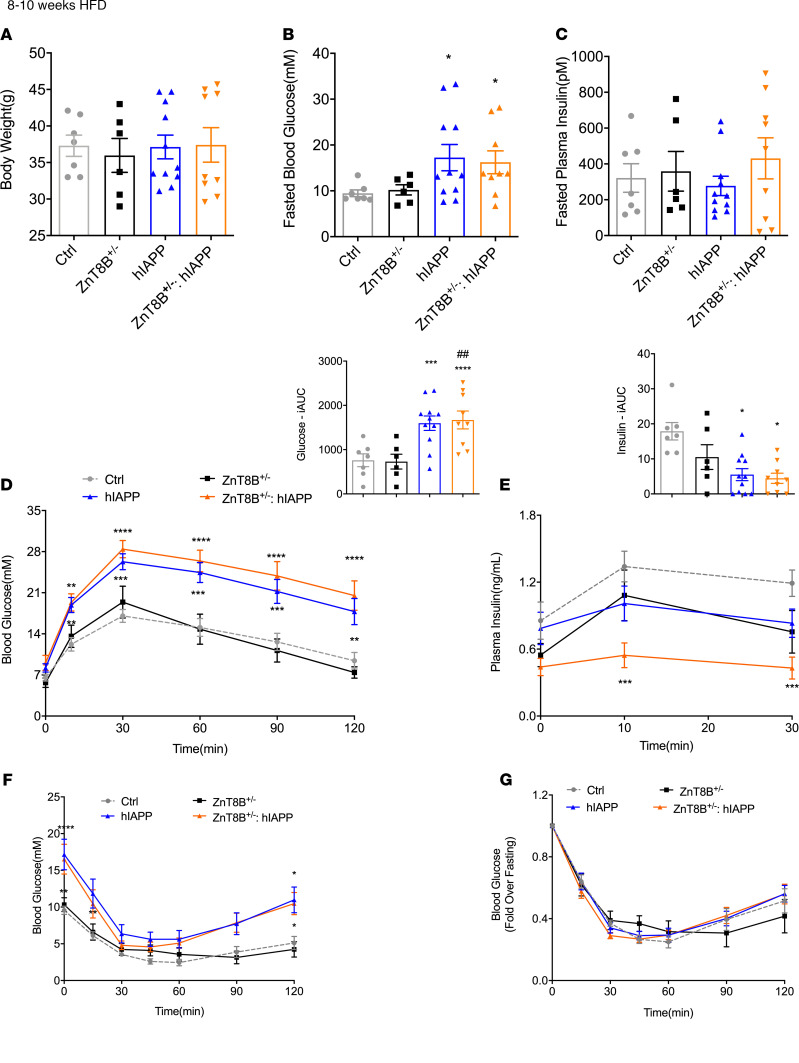
ZnT8B^+/–^ hIAPP mice fed a HFD are glucose intolerant with impaired insulin secretion. (**A**) Body weight, (**B**) 5-hour fasted blood glucose, (**C**) 5-hour fasted plasma insulin, (**D**) OGTT and incremental area under the glucose curve, (**E**) plasma insulin during OGTT and incremental area under the insulin curve. (**F**) Insulin tolerance test (ITT) with (**G**) expression of blood glucose as fold change over fasting. (**D**–**F**) *P < 0.05, ***P < 0.001, ****P < 0.0001, all groups compared with controls. **^##^***P* < 0.01, all groups compared with ZnT8B^+/–^. Data are represented as mean ± SEM. (**A**–**G**) Control (*n* = 7), ZnT8B^–/–^ (*n* = 6), hIAPP (*n* = 11), and ZnT8B^–/–^ hIAPP (*n* = 9). One-way ANOVA with Tukey’s adjustment was used in **A**–**E**. Two-way ANOVA with Dunnett’s correction was used in **D**–**G**. The same control and hIAPP animals from Figure 3 were used in these experiments.

**Figure 7:**
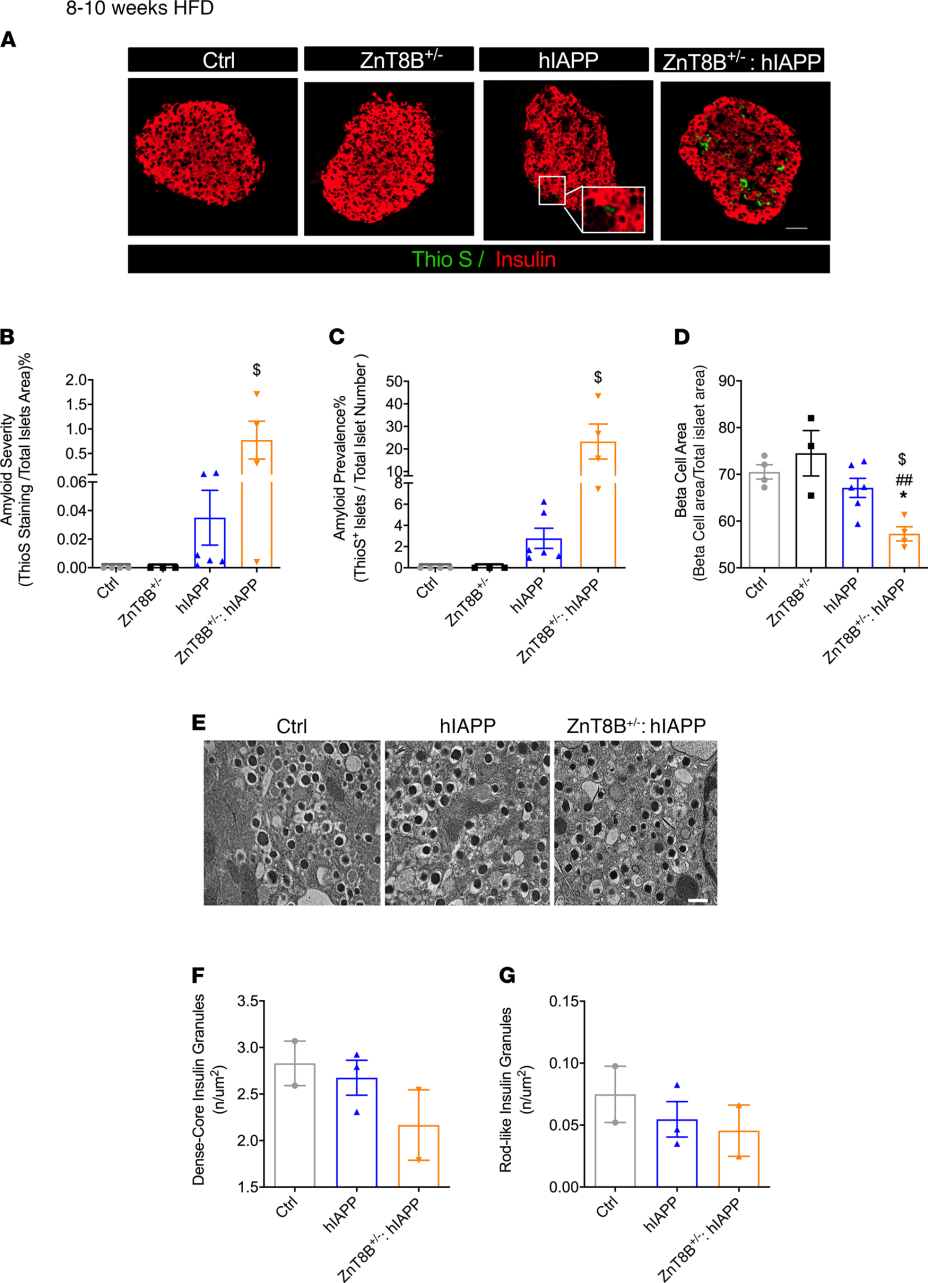
β Cell ZnT8 haploinsufficient hIAPP mice have increased islet amyloidogenesis and β cell loss but normal β cell morphology. (**A**) Thioflavin S staining (scale bar: 50 μm) with quantification of (**B**) amyloid severity, (**C**) amyloid prevalence, and (**D**) β cell area. (**E**) Transmission electron microscopy on isolated islets (scale bar: 500 nm). (**F**) Quantifications of the number of dense-core granules and (**G**) rod-like insulin granules. Data are represented as mean ± SEM. (**A**–**D**) Control (*n* = 4), ZnT8B^–/–^ (*n* = 3), hIAPP (*n* = 6), and ZnT8B^–/–^ hIAPP (*n* = 4). (**E** and **G**) Control (*n* = 2), hIAPP (*n* = 3), and ZnT8B^+/–^ × hIAPP (*n* = 2). *P < 0.05, all groups compared with control; **^##^***P* < 0.01, ZnT8B^+/–^ hIAPP compared with ZnT8B^+/–^. ^$^*P* < 0.01, ZnT8B^+/–^ hIAPP compared with hIAPP. One-way ANOVA with Tukey’s adjustment was used in **B**–**D**, **F**, and **G**.

Heterozygous β cell–specific ZnT8-KO (ZnT8B^+/–^) hIAPP mice showed hyperglycemia and glucose intolerance at 8–10 weeks of HFD feeding, similar to hIAPP mice (Figure 6, A–D). They did show further impairment in in vivo insulin secretion (Figure 6E) and remained insulin sensitive at this age (Figure 6, F and G). Similar to ZnT8B^–/–^ hIAPP, ZnT8B^+/–^ hIAPP mouse islets showed increased amyloid severity and prevalence compared with hIAPP islets and had significantly reduced β cell area compared with control, ZnT8B^+/–^, and hIAPP islets (Figure 7, A–D). However, they showed no change in the number of dense-core and rod-like insulin granules compared with the controls (Figure 7, E and F).

## Discussion

In recent years, ZnT8 has attracted much attention because of its link to T2D through GWAS (32). However, the importance of ZnT8 activity to glucose homeostasis remains debatable. Regarding loss of ZnT8 activity, some studies show the deterioration of β cell function, whereas others suggest no net effect and others suggest improvement (15, 18, 19, 22, 23). With hIAPP-derived amyloid being a significant contributor to β cell toxicity, islet function, and diabetes pathogenesis, it provided an excellent foundation for us to examine the effects of altering ZnT8 activity specifically in β cells and to examine the contribution of ZnT8 to pathological aggregation of this important resident of the insulin granule. Our study showed that the presence of hIAPP in β cells lacking ZnT8 exacerbated amyloidogenesis and β cell dysfunction, strongly suggesting that normal ZnT8 activity is not only important for insulin biosynthesis and β cell function, but also for protecting the β cell from amyloid aggregation (Supplemental Table 1).

The impact on β cell function in HFD-fed ZnT8B^–/–^ hIAPP mice was significant from a young age, leading to differences in insulin secretion and glucose homeostasis compared with mice that expressed hIAPP in the presence of ZnT8. However, this difference was lost as animals aged and the impact on β cell area became similarly significant in hIAPP mice. The absence of ZnT8 is associated with reduced glucose sensing, cell synchronicity with significantly dampened glucose-dependent Ca^2+^ influx, insulin crystallization, and a general defect in insulin packaging, along with impairment in insulin processing in β cells (18, 33). With the presence of hIAPP, our study is the first, to our knowledge, to attempt mimicking the human pathological condition associated with T2D as we try to understand the physiological role of ZnT8 in β cells. Previous mouse models of ZnT8 deficiency lacked the presence of this very important contributor to T2D, including recent studies that used mouse models carrying a human loss-of-function allele that had increased insulin secretory capacity (22, 23). Hyperinsulinemia and impaired insulin processing seen in hIAPP mice lacking ZnT8 suggests that a similar hypersecretion of hIAPP along with impaired hIAPP processing may have contributed to the increased amyloid accumulation in these animals. Defects arising from lack of ZnT8 combined with cell toxicity induced by amyloid aggregates may have led to the severe phenotype observed in these mice.

Reduction in ATP production observed in Figure 5 supports an effect on mitochondria in ZnT8B^–/–^ hIAPP mice. hIAPP is associated with mitochondrial dysfunction, which may be exacerbated in the absence of ZnT8 due to the increased amyloid deposition. In other relevant studies, hIAPP overexpression in β cells induced MTORC1 activation and mitophagy inhibition, leading to an accumulation of damaged and fissioned mitochondria, elevated ROS production, and increased susceptibility to ER stress–induced apoptosis (34). In addition, activity of mitochondrial complex I and IV has been shown to be reduced in the presence of human amylin (35). Another study showed that hIAPP-induced apoptosis of INS-1E cells was associated with the disruption of mitochondrial function as evidenced by ATP depletion, mitochondrial mass reduction, mitochondrial fragmentation, and loss of mitochondrial membrane potential (ΔΨ[m]). Further molecular analysis showed that hIAPP induced changes in the expression of Bcl-2 family members, release of cytochrome c and apoptosis-inducing factor from mitochondria into cytosol, activation of caspases, and cleavage of poly (ADP-ribose) polymerase (36). Additionally, we observed that ZnT8B^–/–^ islets from HFD-fed mice had impaired mitochondria function compared with control islets. ZnT8 deletion in pancreatic β cells reduced cytosolic and granular free zinc levels (15, 33, 37). Decreased zinc levels in the cytosol have been shown to significantly inactivate the mitochondrial biogenetic pathway, reduce mitochondrial DNA replication, and reduce the expression of mitochondrial complex proteins (38, 39). Furthermore, zinc deficiency has been shown to increase ROS production and reduce ATP production (40). Therefore, ZnT8 deficiency in the presence of amyloid aggregation would exacerbate mitochondrial dysfunction in ZnT8B^–/–^ hIAPP islets.

Since the reported loss of ZnT8 function is caused by haploid mutations, we also examined the effects of ZnT8 heterozygous KO in hIAPP-transgenic animals. These mice showed increased amyloid aggregation resulting in exaggerated metabolic imbalances. This is in agreement with a study that showed ZnT8 haploinsufficiency in MIN6 cells led to reduced cellular zinc, cellular insulin, cell proliferation, and survival (41) and a study that showed ZnT8 overexpression and supplementation in human islets protect against palmitate-induced reductions in glucose-stimulated insulin secretion and cell death, respectively (42). In contrast, a recent study showed that heterozygous deletion of ZnT8 in young mice and partial knockdown of ZnT8 in adult mice had no effect on glucose tolerance, suggesting that haploinsufficiencies are likely tolerated because of compensatory adaptations during development (43). The study also suggested that ZnT8 haploinsufficiency protects against diet-induced obesity depending on the genetic background (43).

However, our data suggest that loss of ZnT8 function specifically in β cells increased T2D risk and was not protective. Flannick et al. reported 12 rare variants examined primarily in a European population and with allele frequencies of much less than 1%. Only approximately 350 carriers were found in about 150,000 genotyped individuals. Assessing *SLC30A8* haploinsufficiency in controls versus individuals with T2D suggested that rare loss-of-function variant carriers had reduced T2D risk. On the one hand, the discrepancy seen in humans versus mice could be due to distinct pathology variations between species and the highly pleiotropic effects of Zn^2+^ on β cell function. Rutter et al. suggested that in humans, the relationship between ZnT8 activity and diabetes risk may follow a complex (e.g., bell-shaped) dose response (44). Thus, a relatively small reduction in ZnT8 activity (in the form of the Arg325Trp variant) may be harmful, whereas a large reduction in activity (loss of function) may be protective, the latter by improving insulin secretion greater than insulin clearance (44). However, whether the Arg325 variant has lower zinc transport activity remains contradictory (15, 24–26). In the present study, we observed a similar direction as previous ZnT8B-KO studies showing that reduced ZnT8 activity worsened pancreatic β cell function (19, 20) and associated it with accelerated amylogenesis. Future experiments need to examine hIAPP aggregation in humans presented with ZnT8 polymorphism and loss-of-function mutations to see whether the impact remains comparable. On the other hand, the discrepancy in part may also be explained by the fact that we manipulated β cell–specific ZnT8 activity, and others have examined the global impact of ZnT8 haploinsufficiency or KO. This may suggest that ZnT8 has roles in other tissues. In fact, we previously showed significant differences between whole-body and β cell–specific KO of ZnT8 in mice on a HFD (19). Whole-body ZnT8-KO mice became obese and diabetic, whereas the β cell–specific KO mice were glucose intolerant and had similar weight gain compared with controls. The whole-body KO mice also showed a significant reduction in glucagon secretion. Interestingly, glucagon secretion was unaltered in the β cell–specific KO mice, but when isolated islets were incubated with exogenous zinc, they responded with a reduction in glucagon secretion (45). We have indeed previously shown α cell expression of ZnT8, specifically associated with the glucagon granules (18). In addition, overexpression of ZnT8 in α cells was shown to increase granular zinc and inhibit glucagon secretion (46). A recent study has also reported ZnT8 expression in enteroendocrine cells and a previously unknown role in regulating peripheral 5-hydroxytryptamine biosynthesis, which promotes lipid deposition and obesity (47). Therefore, the discrepancy observed between the current study and in human carriers of the ZnT8 loss-of-function alleles may be due in part to the contribution from extra–β cell tissue/cells. In addition, a recent study suggested that mice with complete deletion of ZnT8 may have an early impairment of glucose tolerance at a young age that is transient due to adaptation during development, leading to restoration of glucose tolerance at an older age (43). In the current study, we observed that ZnT8B^–/–^ mice on a chow diet showed similar glucose tolerance compared with controls at 14 weeks of age. Previously, we reported that ZnT8B^–/–^ mice on a chow diet had impaired glucose homeostasis at 6 weeks of age (18). ZnT8B^–/–^ mice show variable tolerance to glucose at different ages, first reported by Mitchell et al (33). Chow-fed ZnT8B^–/–^ male mice were only glucose intolerant compared with controls at the age of 10 weeks but not at 14 weeks. Additionally, the different backgrounds and diets of the mice may have also contributed to the variable observations in studies. Mice used in the present study were at a 50% FVB and 50% C57 strain on the D12450J diet (20% protein, 10% fat, 70% carbohydrates), whereas a pure C57 strain with a Teklad 2018 diet (24% protein, 18% fat, 58% carbohydrates) was used by Wijesekara et al. (18).

Pancreatic islet β cell–specific/selective KO mouse models are instrumental in studying the effects of T2D susceptibility genes on β cell function. However, each model has its own strengths and shortcomings that need to be considered (48–50). In the present study, the RIP-Cre (51) mouse model was used to generate ZnT8B^–/–^ hIAPP mice. There are potential impediments with the use of this mouse model, imposing some possible limitations. First, Cre expression in the hypothalamus and off-target recombination in a subset of hypothalamic neurons is possible (48). However, we have previously shown that ZnT8 is not expressed in the hypothalamus (52), so this is not likely an issue here. Second, Brouwers et al. reported a possible influence of the hGH mini-gene, present in Pdx1-Cre-Late and Rip-Cre constructs on islet function (49). In that study, hGH mini-gene mice were associated with detectable levels of islet immunoreactive hGH, increased β cell mass, and increased insulin content in the Pdx1-Cre-Late line (49). In the current study, although hGH transcripts were detected in Cre^+^ mouse islets, hGH protein was not (Supplemental Figure 1). This is in line with a study in which hGH transcripts were detected but without any appreciable hGH peptide (53). Newer Ins1-Cre lines generated by gene knockin strategies, with potentially fewer off-target effects, offer a favorable alternative to RIP-Cre (54). However, these newer lines can show variable Cre expression, and thus activity should be carefully monitored over the course of their use (50). Alternative Cre-lines with suitable controls will be considered in our future studies on ZnT8 in β cells to complement the studies described here.

In conclusion, the functional role of ZnT8 is complicated, and before we can establish ZnT8 as a target for treatment at maintaining insulin secretory capacity, more research geared toward understanding cell-specific functions of ZnT8 and functional interaction between tissues/cells is required. Nonetheless, this study confirmed that loss of ZnT8 in pancreatic β cells is deleterious, leading to loss of both β cell area and function in part due to the toxicity induced by hIAPP aggregates. Therefore, ZnT8 is indeed crucial for the maintenance of normal glucose homeostasis, especially when metabolically challenged (e.g., in obesity).

## Methods

### Mice.

Pancreatic β cell–specific ZnT8-KO mice (C57BL/6J-Ins2-Cre^+^ ZnT8^loxp/loxp^) on a C57BL/6 background were generated as previously described (18). RIP was chosen to drive Cre expression in β cells for deletion of the ZnT8 gene because we did not observe ZnT8 expression in the hypothalamus and did not observe any β cell toxicity associated with this line (52). hIAPP-expressing (FVB/N-Tg[Ins2-IAPP]RHFSoel/J) mice on an FVB background were obtained from The Jackson Laboratory and have been previously described (28). Briefly, hemizygous hIAPP-transgenic mice were crossed with pancreatic β cell–specific ZnT8-null mice (ZnT8B^–/–^) to generate Cre^+^ ZnT8B^loxp/+^ hIAPP and ZnT8B^loxp/+^ mice, which were crossed together to generate ZnT8B^–/–^ hIAPP and ZnT8B^+/–^ hIAPP experimental mice. Control (ZnT8^loxp/loxp^), ZnT8B^–/–^, ZnT8B^+/–^, and hIAPP mice from the same litters as experimental mice were used as controls (Figure 1, A and B). The genetic background of all mice was 50% FVB and 50% C57. Mice were fed a HFD containing 60% fat (Research Diets, D12492) or a matched-chow diet containing 10% fat (Research Diets, D12450J) starting at 4 weeks of age. Experiments were performed after 8–10 or 20–22 weeks on the diet at 12–14 or 24–26 weeks of age. All experiments were performed in male mice.

### OGTT and insulin tolerance test.

For the OGTT, after a 16-hour fast, glucose (1.5 g/kg for HFD, 2 g/kg for chow) was given by oral gavage. Blood glucose collected from the tail vein was measured using a Contour Next glucometer (Bayer). For the insulin tolerance test, after a 5-hour fast, insulin (1 IU/kg for HFD, 0.5 IU/kg for chow) was given by i.p. injection. Blood glucose collected from the tail vein was measured using a glucometer (18).

### Plasma insulin and proinsulin.

Blood samples from the tail vein were collected in EDTA-coated microvettes (Sarstedt) at 0, 10, and 30 minutes during OGTT and after 5-hour fasting (18). Plasma was separated from whole blood by centrifugation at 4226*g* for 10 minutes at 4°C. Insulin and proinsulin were analyzed using mouse ultrasensitive insulin ELISA (ALPCO).

### Thioflavin S staining.

Paraffin-embedded sections close to the middle of the pancreata (representing the largest pancreatic area of the pancreas) were cut at 100 μm apart at 2 different levels. Sections were immunostained with polyclonal guinea pig anti-insulin (1:200) (Dako) overnight at 4°C. Immunofluorescence staining was carried out with Alexa Fluor 647 goat anti–guinea pig (1:200; Invitrogen, Thermo Fisher Scientific) secondary antibody for 1 hour incubation at room temperature and subsequently stained with 0.5% thioflavin S (MilliporeSigma) for 5 minutes. Images were obtained using a Zeiss confocal microscope or Zeiss Axioscan slide scanner using Zen software. All quantifications were performed by HALO version 2.0.1145.14 (Indica Labs). Amyloid severity was measured as total amyloid area per total islet area, and amyloid prevalence was analyzed by total number of islets positive for thioflavin S detected as a percentage of the total number of islets. β Cell area was quantified as total islet insulin-positive staining per total islet area. More than 50 islets per sample were analyzed.

### Islet isolation and glucose-stimulated hormone secretion.

Islets were isolated from mouse pancreata and glucose-stimulated hormone secretion studies were carried out as previously described using 2.8 and 11.1 mM of glucose (55). Secreted insulin was quantified by homogenous time-resolved fluorescence assay (Cisbio) and normalized to total DNA.

### Seahorse metabolic flux assay.

Forty to 50 intact islets were seeded per well in the Seahorse XF24 islet plate (Agilent). The OCR of islets was measured at 2 mM glucose (basal) and 11 mM glucose. Islets were sequentially injected with 5 μM oligomycin, 2 μM carbonyl cyanide-4-(trifluoromethoxy) phenylhydrazone with 10 mM methyl pyruvate, and 5 μM rotenone with 5 μM antimycin A. Data were normalized to the basal OCR.

### Calcium imaging.

Islets were incubated in the presence of 2.5 μM Fluo-4 (Life Technologies, Thermo Fisher Scientific) for 1 hour at 37°C in 2 mM glucose-BMHH (125 mM NaCl, 5.7 mM KCl, 2.5 mM CaCl_2_, 1.2 mM MgCl_2_, 10 mM HEPES, and 0.1% BSA; pH 7.4). Islets were subsequently loaded into a custom-built microfluidic device (56) and imaged with a Zeiss LSM710 confocal microscope using the 63×/1.4 NA oil immersion objective lens with heated stage set to 37°C. The islets were under flow (200 μL/h) with 2 mM glucose-BMHH for 10 minutes prior to imaging. Fluo-4 was imaged using the 488 nm laser line and a 493–622 nm bandpass filter with a pinhole size of 89.85 μm. Images consisted of 512 × 512 pixels sized at 0.26 μm/pixel and were taken with a pixel dwell time of 1.576 μs. Time series imaging was sequentially performed on islets in 2 mM (2.5 minutes) and 8 mM (8 minutes) glucose with an incubation period of 10 minutes between glucose treatments. We chose 8 mM glucose to slow the Ca^2+^ activity, which allowed us to accurately measure the oscillation frequency and correlation between cells using a confocal microscope.

### Dithizone staining.

Isolated islets were cultured overnight and stained with 100 μg/mL dithizone (MilliporeSigma) in PBS solution at room temperature for 10 minutes (15). Color images of islets were acquired at 10× magnification via a Leica DMi8 inverted microscope supplied with a Leica DFC9000 microscope camera. Quantification of dithizone staining was performed by converting RGB value to Hue, thereby making the measurement independent of the signal intensity (57). ΔHue was calculated by subtracting the Hue of unstained islets.

### Transmission electron microscopy.

Mouse islets were prepared as previously described, and images were acquired using Talos L120C (Thermo Fisher Scientific) (19). Dense-core granules and rod-like granules were manually counted and quantified (19). Ten cells at 10 areas of the grid per mouse were analyzed.

### Statistics.

Data are presented as mean ± SEM. Unpaired 2-tailed Student’s *t* test, 1-way ANOVA, or 2-way ANOVA was applied to assess statistical significance. Posttest comparisons were performed using Dunnett’s and Tukey’s tests. *P* values less than 0.05 were regarded as statistically significant.

### Study approval.

All animal experiments were approved by the Animal Care Committee of the University of Toronto.

## Author contributions

JX designed the experiments and acquired, analyzed, and interpreted the data and contributed to writing the paper. NW drafted the manuscript and provided substantial contributions to the experimental design and interpretation of the data. RR contributed to Figure 1D and Figure 5D. DA contributed to Supplemental Figure 1, G and H. YS contributed to the quantification of the data in Figure 4, F and G, and Figure 7, F and G. AP and AW contributed to the genotyping PCR; AB contributed to the acquisition of the data; YL, LM, JVR, PEF, FFD, CH, and MBW provided contributions to the conception of the study and interpretation of the data. All authors revised and approved the final version of the manuscript. CH and MBW are the guarantors of this paper.

## Supplementary Material

Supplemental data

## Version 1 05/24/2021

Electronic publication

## References

[1] Foster MC (1993). Elemental composition of secretory granules in pancreatic islets of Langerhans. Biophys J.

[2] Dodson G (1998). The role of assembly in insulin’s biosynthesis. Curr Opin Struct Biol.

[3] Saxena R (2007). Genome-wide association analysis identifies loci for type 2 diabetes and triglyceride levels. Science.

[4] Scott LJ (2007). A genome-wide association study of type 2 diabetes in Finns detects multiple susceptibility variants. Science.

[5] Zeggini E (2007). Replication of genome-wide association signals in UK samples reveals risk loci for type 2 diabetes. Science.

[6] Steinthorsdottir V (2007). A variant in CDKAL1 influences insulin response and risk of type 2 diabetes. Nat Genet.

[7] Omori S (2008). Association of CDKAL1, IGF2BP2, CDKN2A/B, HHEX, SLC30A8, and KCNJ11 with susceptibility to type 2 diabetes in a Japanese population. Diabetes.

[8] Ng MC (2008). Implication of genetic variants near TCF7L2, SLC30A8, HHEX, CDKAL1, CDKN2A/B, IGF2BP2, and FTO in type 2 diabetes and obesity in 6,719 Asians. Diabetes.

[9] Hu C (2010). Variants from GIPR, TCF7L2, DGKB, MADD, CRY2, GLIS3, PROX1, SLC30A8 and IGF1 are associated with glucose metabolism in the Chinese. PLoS One.

[10] Hertel JK (2008). Genetic analysis of recently identified type 2 diabetes loci in 1,638 unselected patients with type 2 diabetes and 1,858 control participants from a Norwegian population-based cohort (the HUNT study). Diabetologia.

[11] Lee YH (2008). Association between polymorphisms in SLC30A8, HHEX, CDKN2A/B, IGF2BP2, FTO, WFS1, CDKAL1, KCNQ1 and type 2 diabetes in the Korean population. J Hum Genet.

[12] Staiger H (2007). Polymorphisms within novel risk loci for type 2 diabetes determine beta-cell function. PLoS One.

[13] Kirchhoff K (2008). Polymorphisms in the TCF7L2, CDKAL1 and SLC30A8 genes are associated with impaired proinsulin conversion. Diabetologia.

[14] Cauchi S (2008). Analysis of novel risk loci for type 2 diabetes in a general French population: the D.E.S.I.R. study. J Mol Med (Berl).

[15] Nicolson TJ (2009). Insulin storage and glucose homeostasis in mice null for the granule zinc transporter ZnT8 and studies of the type 2 diabetes-associated variants. Diabetes.

[16] Pound LD (2009). Deletion of the mouse Slc30a8 gene encoding zinc transporter-8 results in impaired insulin secretion. Biochem J.

[17] Lemaire K (2009). Insulin crystallization depends on zinc transporter ZnT8 expression, but is not required for normal glucose homeostasis in mice. Proc Natl Acad Sci U S A.

[18] Wijesekara N (2010). Beta cell-specific Znt8 deletion in mice causes marked defects in insulin processing, crystallisation and secretion. Diabetologia.

[19] Hardy AB (2012). Effects of high-fat diet feeding on Znt8-null mice: differences between β-cell and global knockout of Znt8. Am J Physiol Endocrinol Metab.

[20] Tamaki M (2013). The diabetes-susceptible gene SLC30A8/ZnT8 regulates hepatic insulin clearance. J Clin Invest.

[21] Flannick J (2014). Loss-of-function mutations in SLC30A8 protect against type 2 diabetes. Nat Genet.

[22] Dwivedi OP (2019). Loss of ZnT8 function protects against diabetes by enhanced insulin secretion. Nat Genet.

[23] Kleiner S (2018). Mice harboring the human *SLC30A8* R138X loss-of-function mutation have increased insulin secretory capacity. Proc Natl Acad Sci U S A.

[24] Kim I (2011). A low-risk ZnT-8 allele (W325) for post-transplantation diabetes mellitus is protective against cyclosporin A-induced impairment of insulin secretion. Pharmacogenomics J.

[25] Merriman C (2016). Lipid-tuned zinc transport activity of human ZnT8 protein correlates with risk for type-2 diabetes. J Biol Chem.

[26] Carvalho S (2017). Differential cytolocation and functional assays of the two major human SLC30A8 (ZnT8) isoforms. J Trace Elem Med Biol.

[27] Matveyenko AV (2006). Islet amyloid polypeptide (IAPP) transgenic rodents as models for type 2 diabetes. ILAR J.

[28] Janson J (1996). Spontaneous diabetes mellitus in transgenic mice expressing human islet amyloid polypeptide. Proc Natl Acad Sci U S A.

[29] Hull RL (2003). Increased dietary fat promotes islet amyloid formation and beta-cell secretory dysfunction in a transgenic mouse model of islet amyloid. Diabetes.

[30] Kulkarni RN (1999). Tissue-specific knockout of the insulin receptor in pancreatic beta cells creates an insulin secretory defect similar to that in type 2 diabetes. Cell.

[31] Fex M (2007). Rat insulin promoter 2-Cre recombinase mice bred onto a pure C57BL/6J background exhibit unaltered glucose tolerance. J Endocrinol.

[32] Sladek R (2007). A genome-wide association study identifies novel risk loci for type 2 diabetes. Nature.

[33] Mitchell RK (2016). Molecular genetic regulation of Slc30a8/ZnT8 reveals a positive association with glucose tolerance. Mol Endocrinol.

[34] Hernandez MG (2018). Pancreatic β cells overexpressing hIAPP impaired mitophagy and unbalanced mitochondrial dynamics. Cell Death Dis.

[35] Lim YA (2010). Abeta and human amylin share a common toxicity pathway via mitochondrial dysfunction. Proteomics.

[36] Li XL (2011). Involvement of mitochondrial dysfunction in human islet amyloid polypeptide-induced apoptosis in INS-1E pancreatic beta cells: an effect attenuated by phycocyanin. Int J Biochem Cell Biol.

[37] Gerber PA (2014). Hypoxia lowers SLC30A8/ZnT8 expression and free cytosolic Zn2+ in pancreatic beta cells. Diabetologia.

[38] Sun Q (2016). Defect of mitochondrial respiratory chain is a mechanism of ROS overproduction in a rat model of alcoholic liver disease: role of zinc deficiency. Am J Physiol Gastrointest Liver Physiol.

[39] Bahadorani S (2013). Expression of zinc-deficient human superoxide dismutase in Drosophila neurons produces a locomotor defect linked to mitochondrial dysfunction. Neurobiol Aging.

[40] Rajapakse D (2017). Zinc protects oxidative stress-induced RPE death by reducing mitochondrial damage and preventing lysosome rupture. Oxid Med Cell Longev.

[41] Lawson R (2019). ZnT8 haploinsufficiency impacts MIN6 cell zinc content and β-cell phenotype via ZIP-ZnT8 coregulation. Int J Mol Sci.

[42] Lefebvre B (2012). Regulation and functional effects of ZNT8 in human pancreatic islets. J Endocrinol.

[43] Syring KE (2020). Potential positive and negative consequences of ZnT8 inhibition. J Endocrinol.

[44] Rutter GA (2015). SLC30A8 mutations in type 2 diabetes. Diabetologia.

[45] Hardy AB (2011). Regulation of glucagon secretion by zinc: lessons from the β cell-specific Znt8 knockout mouse model. Diabetes Obes Metab.

[46] Solomou A (2016). Over-expression of Slc30a8/ZnT8 selectively in the mouse α cell impairs glucagon release and responses to hypoglycemia. Nutr Metab (Lond).

[47] Mao Z (2019). Deficiency of ZnT8 promotes adiposity and metabolic dysfunction by increasing peripheral serotonin production. Diabetes.

[48] Magnuson MA (2013). Pancreas-specific Cre driver lines and considerations for their prudent use. Cell Metab.

[49] Brouwers B (2014). Impaired islet function in commonly used transgenic mouse lines due to human growth hormone minigene expression. Cell Metab.

[50] Mosleh E (2020). Ins1-Cre and Ins1-CreER gene replacement alleles are susceptible to silencing by DNA hypermethylation. Endocrinology.

[51] Postic C (1999). Dual roles for glucokinase in glucose homeostasis as determined by liver and pancreatic beta cell-specific gene knock-outs using Cre recombinase. J Biol Chem.

[52] Wijesekara N (2009). Zinc, a regulator of islet function and glucose homeostasis. Diabetes Obes Metab.

[53] Lavine JA (2015). Cholecystokinin expression in the β-cell leads to increased β-cell area in aged mice and protects from streptozotocin-induced diabetes and apoptosis. Am J Physiol Endocrinol Metab.

[54] Thorens B (2015). Ins1(Cre) knock-in mice for beta cell-specific gene recombination. Diabetologia.

[55] Lee SC (2009). Uncoupling protein 2 regulates reactive oxygen species formation in islets and influences susceptibility to diabetogenic action of streptozotocin. J Endocrinol.

[56] Silva PN (2013). A microfluidic device designed to induce media flow throughout pancreatic islets while limiting shear-induced damage. Lab Chip.

[57] Tan Y (2018). Streamlining volumetric multi-channel image cytometry using hue-saturation-brightness-based surface creation. Commun Biol.

