# Supplementary material for: Pancreatic β cell–selective zinc transporter 8 insufficiency accelerates diabetes associated with islet amyloidosis
Source: JCI Insight. 2021 May 24;6(10):e143037. doi: 10.1172/jci.insight.143037 (PMC8262350; doi:10.1172/jci.insight.143037)
Supplement: Supplemental data [file jciinsight-6-143037-s288.pdf]

SUPPLEMENTAL METHODS.

Immunohistochemistry staining. Paraffin embedded sections were stained with anti-insulin (DAKO, Carpinteria, CA) or anti-glucagon (Abcam, Toronto, ON) as described previously (1). Images were taken using Aperio ImageScope at 20x magnification. All quantifications were performed by HALO version 2.0.1145.14 (Indica Labs, Corrales, NM).

1. Untereiner A, Abdo S, Bhattacharjee A, Gohil H, Pourasgari F, Ibeh N, et al. GABA promotes beta-cell proliferation, but does not overcome impaired glucose homeostasis associated with diet-induced obesity. FASEB J. 2019;33(3):3968-84.

Supp. Figure. 1. Cre didn't impact glucose homeostasis and hGH protein is not expressed in islets. Oral glucose tolerance test on Chow-fed A) 6 weeks of age in transgenic mice expressing Cre (n=7) and ZnT8 fl/fl (n=6). In vivo assessment of 8-10 weeks HFD-fed hIAPP mice with/without Cre expression on (B) Body weight, (C) 5h fasted blood glucose, (D) Oral glucose tolerance test (OGTT) and (E) Insulin secretion during OGTT.B-E, Cre^{+} hIAPP (n=6) and Cre^{-} hIAPP (n=8). (F)cDNA PCR of hGH on Cre^{+} WT and Cre^{-} ZnT8flox/flox islet samples. (G) Total islet hGH content and (H) total islet insulin content was measured in Ctrl (n=4), $ZnT8B^{-/-}$ (n=4), hIAPP (n=4) and $ZnT8B^{-/-}$: hIAPP(n=4) mouse islets. Human plasma sample was used as positive control for hGH Elisa. The dotted line represents the sensitivity for hGH ELISA (Y=5 pg/ml). One-way ANOVA with Turkey adjustment was used in A, D, E and H. Unpaired T test was B and C. Data was represented as mean±sem.

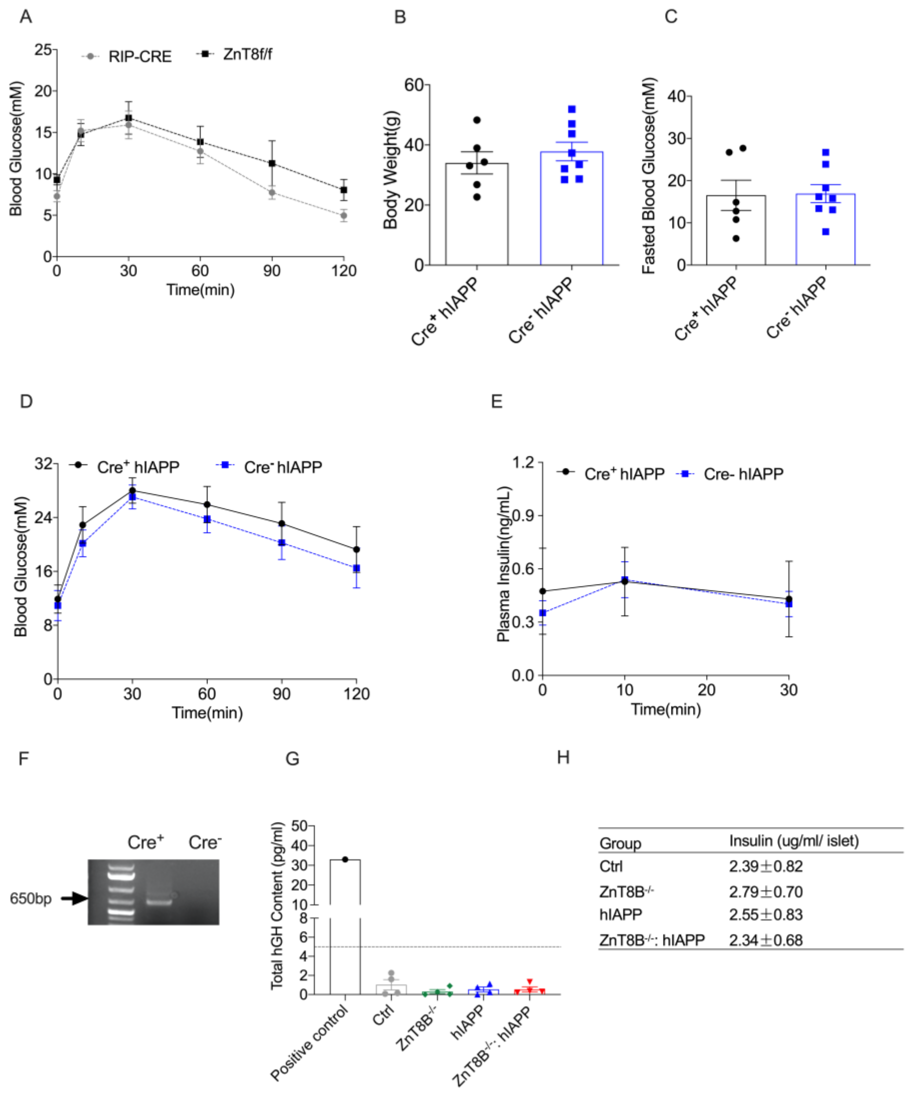

Group	Insulin (ug/ml/ islet)
Ctrl	2.39±0.82
$ZnT8B^{-/-}$	2.79±0.70
hIAPP	2.55±0.83
$ZnT8B^{-/-}$: hIAPP	2.34±0.68

Supp. Figure. 2 ZnT8B$^{-/-}$: hIAPP and hIAPP male mice impaired glucose tolerance and insulin secretion after 20-22 weeks chow or HFD feeding. A) and (F) Body weight, (B) and (G) 5h fasted blood glucose, (C) and (H) 5h fasted plasma insulin, (D) and (I) oral glucose tolerance test (OGTT) with area under the glucose curve, (E) and (J) Insulin secretion during OGTT *P<0.05, **P<0.01, ***P<0.001, ZnT8B$^{-/-}$: hIAPP, hIAPP and ZnT8B$^{-/-}$ vs. Ctrl, $^{##}$ P<0.01, $^{####}$ P<0.0001, ZnT8B$^{-/-}$: hIAPP compared to ZnT8B$^{-/-}$. One-way ANOVA with Turkey adjustment was used in A-J. Two-way ANOVA with Dunnett correction was used in D, I. A-E, Ctrl (n=4). ZnT8B$^{-/-}$ (n=5) hIAPP(n=5) and ZnT8B$^{-/-}$: hIAPP(n=6). F-G, Ctrl (n=9). ZnT8B$^{-/-}$ (n=5) hIAPP(n=7) and ZnT8B$^{-/-}$: hIAPP(n=9).

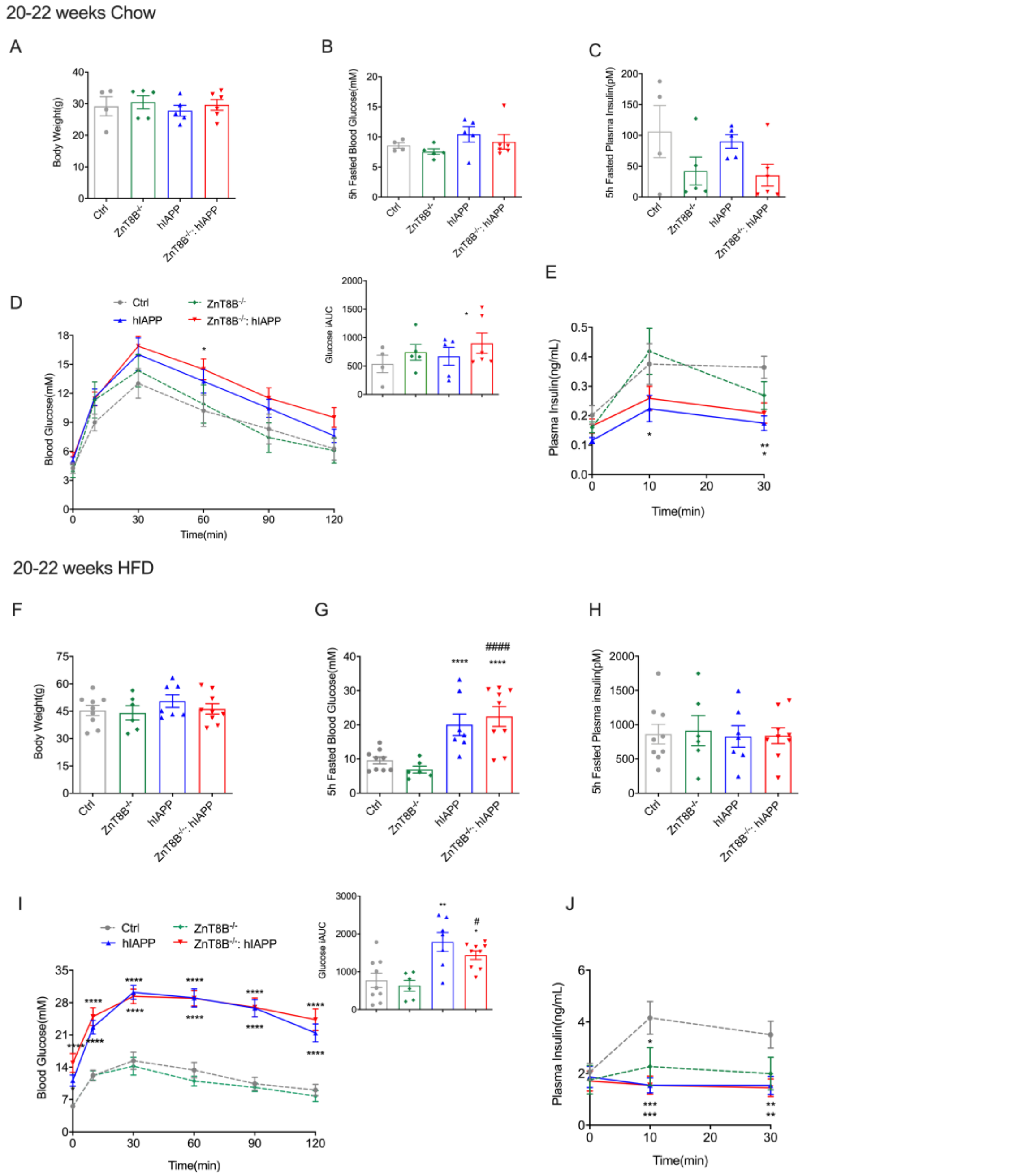

Supp. Figure. 3. 8-10 weeks high fat fed ZnT8B$^{-/-}$: hIAPP mice has normal islet size and number. (A) Histological analysis of insulin staining (n=3 mice/genotype. Scale bar, 50um) with quantification of (B) islet size distribution and (C) average islet size and (D) islet density measured as islet number per the area of pancreatic section. (E) quantification of glucagon positive area per total section area. (F)Histological analysis of glucagon staining (n=3 mice/genotype. Scale bar, 50um) with quantification of glucagon positive area per total section area. One-way ANOVA with Turkey adjustment was used in B, C, D, E and G.

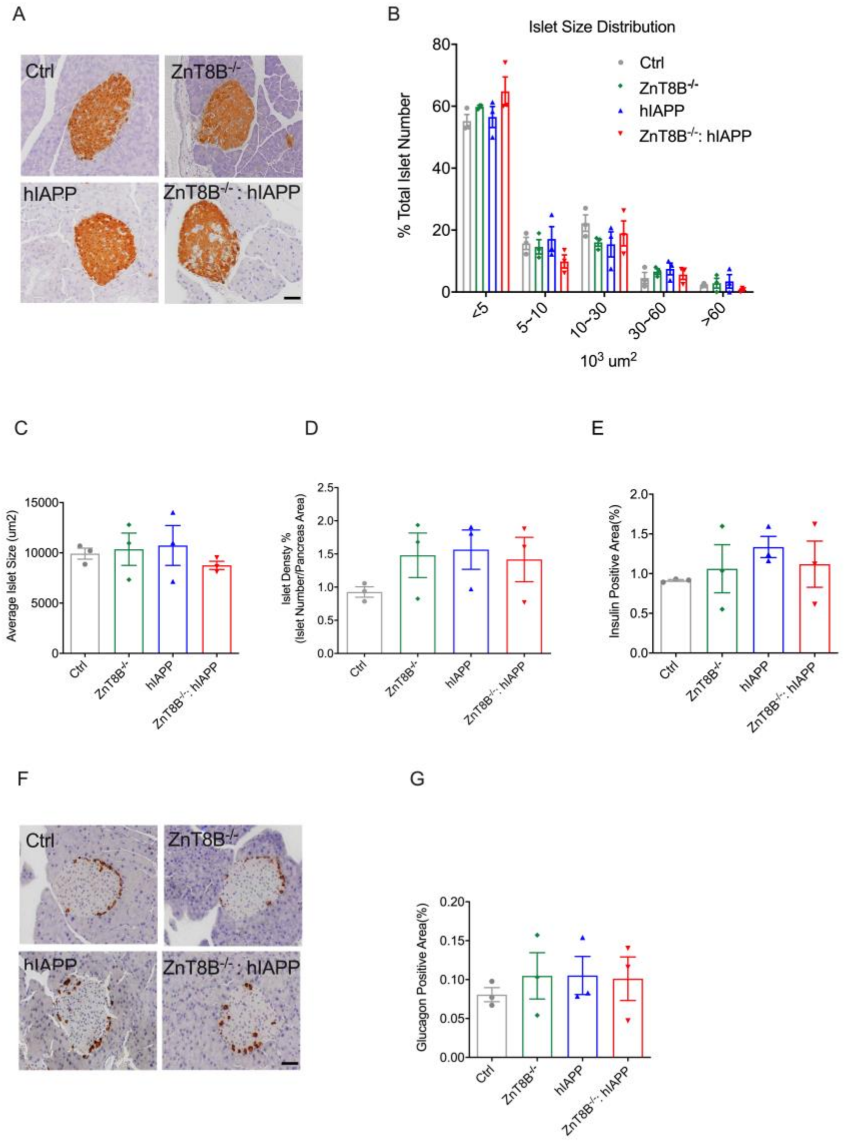

Suppl. Table 1, summarized phenotypes of ZnT8B$^{-/-}$, hIAPP, and ZnT8B$^{-/-}$: hIAPP mice compared to Ctrl under chow and HFD-fed condition.

		ZnT8B$^{-/-}$	hIAPP	ZnT8B$^{-/-}$: hIAPP
	Body weight	-	-	-

8-10 weeks chow	5h fasted blood glucose	-	-	-
	overnight fasted plasma insulin	-	-	-
	OGTT	-	-	Mild impaired
	Insulin during OGTT	-	-	Mild impaired
	Insulin sensitivity	-	-	-
8-10 weeks HFD	Body weight	-	-	-
	5h fasted blood glucose	-	Hyperglycemia	Hyperglycemia
	5h fasted plasma insulin	-	-	hyperinsulinemia
	OGTT	Mild impaired	Glucose intolerance	Severe glucose intolerance
	Insulin during OGTT	-	Impaired insulin secretion	Impaired insulin secretion
	Insulin sensitivity	-	-	-
	Ex vivo GSIS	-	-	Impaired insulin secretion
	Total insulin content	-	-	-
	Amyloid prevalence	NT	Detected	Increased
	Amyloid severity	NT	Detected	Increased
	Beta cell area	-	-	Reduced
	Alpha cell area percentage	-	-	-
	Islet size distribution	-	-	-
	Islet number	-	-	-
	Islet density	-	-	-
	Dense-core ISG	Reduced	-	Reduced

	Rod-like ISG	Increased	-	Reduced

*, “-” represented no change versus Ctrl; NT, not detected.
